# Mechanisms and regulations of ferroptosis

**DOI:** 10.3389/fimmu.2023.1269451

**Published:** 2023-10-06

**Authors:** Xu-Dong Zhang, Zhong-Yuan Liu, Mao-Sen Wang, Yu-Xiang Guo, Xiang-Kun Wang, Kai Luo, Shuai Huang, Ren-Feng Li

**Affiliations:** Departments of Hepatobiliary and Pancreatic Surgery, The First Affiliated Hospital of Zhengzhou University, Zhengzhou, China

**Keywords:** ferroptosis, iron metabolism, lipid metabolism, lipid peroxidation, autophagy

## Abstract

Regulation of cell mortality for disease treatment has been the focus of research. Ferroptosis is an iron-dependent regulated cell death whose mechanism has been extensively studied since its discovery. A large number of studies have shown that regulation of ferroptosis brings new strategies for the treatment of various benign and malignant diseases. Iron excess and lipid peroxidation are its primary metabolic features. Therefore, genes involved in iron metabolism and lipid metabolism can regulate iron overload and lipid peroxidation through direct or indirect pathways, thereby regulating ferroptosis. In addition, glutathione (GSH) is the body’s primary non-enzymatic antioxidants and plays a pivotal role in the struggle against lipid peroxidation. GSH functions as an auxiliary substance for glutathione peroxidase 4 (GPX4) to convert toxic lipid peroxides to their corresponding alcohols. Here, we reviewed the researches on the mechanism of ferroptosis in recent years, and comprehensively analyzed the mechanism and regulatory process of ferroptosis from iron metabolism and lipid metabolism, and then described in detail the metabolism of GPX4 and the main non-enzymatic antioxidant GSH *in vivo*.

## Introduction

Currently there are two types of cell death, according to the most recent recommendations of the 2018 Committee on Nomenclature of Cell Death: accidental cell death (ACD) and regulatory cell death (RCD) (1). ACD is a biologically uncontrolled process triggered by unexpected noxious stimuli that are beyond the regulatory capacity of the cell (2). RCD, also known as programmed cell death (PCD), is a type of cell death process regulated by specific signaling pathways in cells and has biological functions (3). RCD are further subdivided into apoptotic and non-apoptotic types, among which non-apoptotic types are further segmented into ferroptosis, necroptosis, pyroptosis, autophagy, cuproptosis and alkaliptosis, etc. (1, 4), each of which has distinct signal induction, molecular regulation, and disease-causing effects. The discovery of RCD has facilitated progress in the treatment of benign and malignant disorders.

Ferroptosis is caused by the redox imbalance between oxidation and antioxidant, which is driven by the abnormal expression of a variety of redox active enzymes. It is characterized by increased lipid peroxides levels and iron overload (5, 6). Lipid peroxides are highly reactive molecules that can damage cellular components, including lipins, proteins, and DNA, leading to cell death (7). Different from the morphological changes of classic apoptotic cells, such as cell shrinkage, nuclear volume reduction, and highly condensed chromatin to form apoptotic bodies, ferroptosis has its unique morphological characteristics, the density of the mitochondrial membrane increases, the size shrinks, and the structure of the mitochondrial inner cristae disappears (8). Ferroptosis involves complex signaling pathways, since its identification in 2012 (8), it has been a subject of intense study. Numerous studies reveal that ferroptosis is a key regulator in a wide variety of diseases, including cancers, ischemia-reperfusion injury, renal failure, neurological disorders, and hematological conditions (9, 10).

In 2014, Yang et al. (11) used proteomics to find that GPX4 has a regulatory effect on ferroptosis induced by 12 ferroptosis inducers, thus confirming that GPX4 plays a central role in the regulation of ferroptosis. GPX4 is a selenium-dependent enzyme, and it contains a selenium-bound amino acid residue that is essential for its reductive activity (12). GPX4 can reduce lipid peroxides and plays a crucial function in prohibiting lipid peroxidation (13). Actually, GSH is not directly involved in the reduction of lipid peroxides, but it does maintain the reducibility of selenocysteine residues within the active site of GPX4 (13). Cysteine is the rate-limiting precursor of GSH synthesis, and intracellular cysteine content is limited, necessitating the transport of extracellular cystine for intracellular cysteine synthesis (14). Solute carrier family 7 member 11 (SLC7A11) functions as a cystine transporter and thus acting a significant purpose in ferroptosis. Current research has shown that a variety of genes and pharmaceuticals can modulate ferroptosis by targeting the GPX4, GSH or SLC7A11 (15–17).

Due to the fact that ferroptosis entails dysregulation of lipid metabolism, iron metabolism, the oxidation system and the antioxidant system, we reviewed the current research on ferroptosis from these three perspectives. In this publication, the mechanism of ferroptosis was summarized in detail, providing a foundation for future research. However, our understanding of the molecular mechanism of ferroptosis and the usage of ferroptosis in physiological and pathological processes is still limited, constraining the clinical transformation of ferroptosis and highlighting the need for future research in this area.

## Iron metabolism

Iron occurs primarily as two different forms in the human body: trivalent iron ion (Fe^3+^) and ferrous ion (Fe^2+^). Iron is absorbed and used in the human body as Fe^2+^ (18), and it is transported as Fe^3+^ (19). Iron comes in two main forms in food: heme and nonheme iron. The hemoglobin and myoglobin of meat are the main sources of heme iron, which is Fe^2+^. Nonheme iron mainly comes from botany, which is Fe^3+^. Vitamin C and hydrochloric acid can convert nonheme to Fe^2+^, making it easier to absorb (20). The upper jejunum and duodenum are the primary sites of iron absorption from food. Duodenal cytochrome b (*DcytB*) is highly expressed in the brush membrane of duodenal epithelial cells, which can convert Fe^3+^ into Fe^2+^ (21). As a ferric iron reductase, *DcytB* plays an irreplaceable role in iron absorption. However, no research has revealed whether *DcytB* has a regulatory effect on ferroptosis. Patients with hereditary hemochromatosis show progressive iron overload, and most patients have missense mutations in the Hfe gene (C282Y). Herrmann et al. (22) used the *Hfe* knockout mouse model to prove that increased expression of *Dcytb* plays a role in the pathogenesis of iron overload in *Hfe* deficiency. Therefore, we conjecture that *DcytB* can also regulate ferroptosis, but there hasn’t been any direct study to prove the link between the two.

Then, Fe^2+^ is taken into the cell via the divalent metal transporter 1 (*DMT1*, the major transmembrane transporter of divalent metal cations into cells) at the lumen side of tiny intestinal epithelial cells (23). In addition to transporting Fe^2+^, *DMT1* can also transport Zn^2+^, Mn^2+^, Co^2+^, Cd^2+^, Cu^2+^, Ni^2+^, and Pb^2+^. *DMT1* is one of the the principal iron transporters responsible for Fe^2+^ uptake in the majority of cell types. Therefore, by regulating the expression of *DMT1*, ferroptosis can be regulated. Iron is essential for normal neurological function, but current studies have found that excess iron in nerve cells is strongly associated with several neurodegenerative diseases. Multiple or prolonged exposure to general anesthesia leads to cognitive deficits in patients, which have been found to be associated with iron overload due to abnormal activation of *DMT1* in the brain (24). In addition, isoliquiritigenin, as a classic hepatoprotective drug, induces ferroptosis in hepatic stellate cells by promoting the expression of *DMT1*, and ultimately alleviates liver fibrosis (25). The above findings demonstrate the essential role of *DMT1* in regulating iron metabolism and ferroptosis.

After the absorption of Fe^2+^ in the small intestine, some Fe^2+^ can synthesize ferritin in intestinal mucosal epithelial cells, and the other part enters the blood circulation. Ferritin is considered a major iron-storage protein because it can accumulate in large amounts iron ion (26). The ferritin family consists of 4 subfamilies, including the canonical ferritin (FTN), the heme-containing bacterioferritin (BFR), DNA-binding proteins from starved cell (DPS), and, more recently, the discovery of the encapsulated ferritin (27, 28). It exists mainly in the cytoplasm and a small amount in the nucleus, mitochondria and circulating plasma. In humans, ferritin is the canonical ferritin, FTN. BFR and DPS are limited to prokaryotes. FTN is composed of two subunits of 19 kDA ferritin light chain (L) and 21 kDA ferritin heavy chain (H). Poly (RC)-binding proteins (*PCBPs*) and nuclear receptor coactivator 4 (*NCOA4*) have been identified as essential regulators of ferritin, which in turn play a role in ferroptosis (29, 30)..

Then, Fe^2+^ travels via ferroportin (FPN, the only known cellular iron exporting protein) on the basolateral membrane side to the blood circulation (31). FPN is highly express in duodenal enterocytes, liver kupffer cells and splenic red pulp macrophages and plays an extremely important role in the exportation of iron. In Alzheimer’s disease, loss of FPN leads to excess iron in neurons, which eventually promotes disease progression through ferroptosis (32). Hepcidin-FPN rigorously regulates serum iron levels. When iron is deficient *in vivo*, hepcidin expression decreases, allowing iron to be delivered to plasma via FPN (33). The role of hepcidin in ferroptosis will be described in detail later.

**Figure 1:**
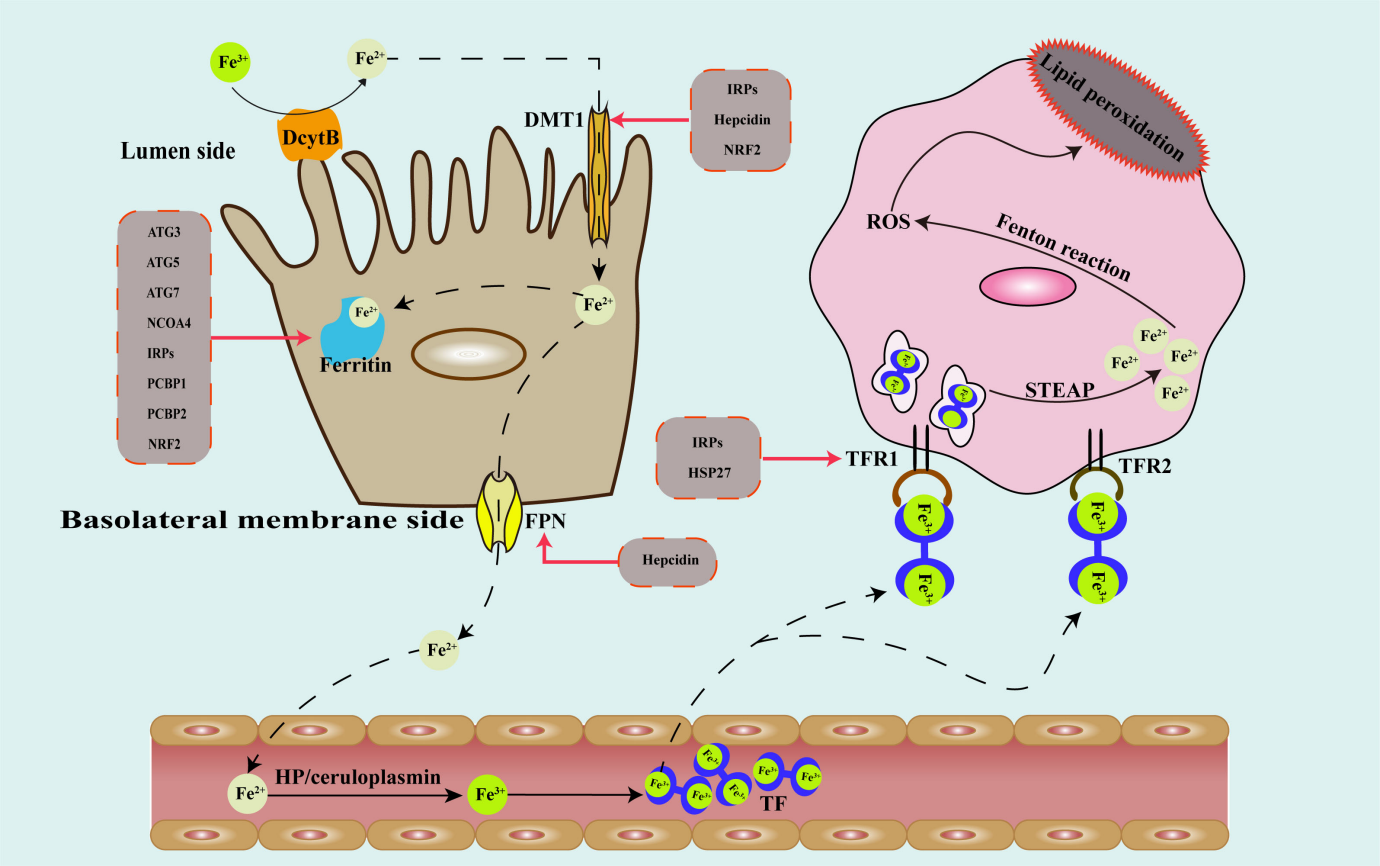
Schematic diagram of the metabolic pathway of iron in the human body and regulatory targets of genes that modulate iron metabolism. DcytB, duodenal cytochrome b; DMT1, divalent metal transporter 1; FPN, ferroportin; HP, hephaestin; TF, transferrin; TFR1, transferrin receptor 1; TFR2, transferrin receptor 2; STEAP3, six-transmembrane epithelial antigen of prostate 3; ROS, reactive oxygen species; ATG, autophagy associated gene; NCOA4, nuclear receptor coactivator 4; IRPs, iron response element binding proteins; PCBP, poly(rC)-binding proteins; HSP, heat shock protein; NRF2, nuclear factor erythroid 2-related factor 2.

Under the effect of hephaestin (HP) or ceruloplasmin, Fe^2+^ entering the bloodstream is converted into Fe^3+^, which combines with transferrin and is transported to the tissues. Each transferrin can carry two Fe^3+^, making it the primary iron transporter (34). Gao et al. discovered that consumption of transferrin in fetal bovine serum (FBS) can weaken ferroptosis, and that this effect was only seen in iron-carrying transferrin (35). Afterwards, transferrin attaches to transferrin receptor 1 (TFR1) and transferrin receptor 2 (TFR2) on the cell surface of iron-deficient cells and enters the cell in a controlled manner (36, 37). TFR1 is widely expressed on the surface of a variety of cells, and binds to transferrin carrying iron, which then enters the cell through endocytosis, and Fe^3+^ that enters the cell is reduced to Fe^2+^ by six-transmembrane epithelial antigen of prostate 3(*STEAP3* metal reductase) (38). Unlike TFR1, the expression of TFR2 is more specific. TFR2 is mainly expressed in hepatocytes and interacts with Hfe protein to regulate iron metabolism in the liver (38). TFR1 and TFR2 can also serve as targets for ferroptosis regulation (39, 40). **Figure 1** shows roughly how iron is absorbed, transported, stored and transformed in the body.

As a crucial trace element in the human body, iron plays a crucial part in multiple biological processes. However, iron also has redox-active, it can generate many reactive oxygen species (ROS) through the fenton reaction and other ways (41, 42), further leads to cell damage and death, including ferroptosis. Therefore, iron homeostasis is strictly regulated. In theory, factors that can regulate the absorption, distribution, transport and utilization of iron in the body can regulate ferroptosis.

## Lipid peroxidation

Lipid peroxidation refers to oxidative deterioration of polyunsaturated fatty acids (PUFAs) and lipids (43). It can disrupt cell membranes, lipoproteins, and other structures containing lipids, hence impairing the proper function of cells. PUFAs refer to straight-chain fatty acids with two or more double bonds and 18 to 22 carbon atoms in the carbon chain, which usually separated into omega-3 and omega-6. Members of the omega-3 fatty acid family, mainly alpha-linolenic acid, eicosapentaenoic acid (EPA) and docosahexaenoic acid (DHA). The omega-6 series fatty acids mainly include linoleic acid (LA, octadecadienoic acid) and arachidonic acid (AA, eicosadonic acid). The hydrogen on the methylene group (-CH2-) connected with two double bonds in the PUFAs molecule is more active, so it is more prone to peroxidation (44). Among them, AA and adrenic acid (AdA) are the main substrates of lipid peroxidation in ferroptosis (45). Hydrogen atoms in lipid substances are extracted by active substances to generate lipid free radicals. Then lipid free radicals react with active oxygen to generate lipid peroxy free radicals, which can continue to extract hydrogen atoms from other substances to form lipid peroxides (13). In a study on inflammatory bowel disease, researchers found that dietary supplementation with PUFAs promoted the progression of ferroptosis-associated inflammatory bowel disease in a mouse model (46). Monounsaturated fatty acids (MUFAs) such as oleic acid and palmitoleic acid can effectively inhibit erastin-induced ferroptosis by competing with PUFAs for incorporation into phospholipids (44, 47). This is because MUFAs do not have double Allyl group position, therefore are not simple to peroxidized. Mitochondrial 2,4-dienoyl-CoA reductase (*DECR1*) is one of the key enzymes for PUFAs β-oxidation. Studies have found that in prostate cancer, inhibiting the expression of *DECR1* can inhibit PUFAs β-oxidation and increase the content of PUFAs in cells, thereby enhancing mitochondrial stress and lipid peroxidation (48, 49).

This indicates that fatty acid β-oxidation plays a crucial function in balancing PUFAs and MUFAs. Lipid peroxidation to form lipid peroxidation products such as malondialdehyde (MDA) and 4-hydroxynonenoic (4-HNE). Therefore, people often judge the level of ferroptosis in cells by detecting the levels of MDA and 4-HNE in cells (50).

Enzymatic and non-enzymatic lipid peroxidation are the two forms of lipid peroxidation (51). Non-enzymatic lipid peroxidation is driven mainly by hydroxyl radicals generated by the interaction of Fe^2+^, Cu^2+^, Co^2+^, and other transition metal ions with ROS (52). Despite being a potent oxidizing agent, molecular oxygen does not directly participate in nonenzymatic lipid peroxidation reactions. Lipoxygenases (*LOXs*) is the key enzymes involved in enzymatic lipid peroxidation. There are mainly 6 kinds of *LOXs* in the human body, namely *LOX5*, *LOX12*, *LOX12B*, *LOX15*, *LOX15B* and *LOXE3*. In addition, oxidases such as cytochrome p-450 oxidoreductase (POR) can also promote lipid peroxidation (53). POR is a NADPH-dependent, FMN-containing oxidoreductase. Zou et al. (53) found that POR has a pro-ferroptosis role in different cancer lineages and used systematic lipidomic analysis to demonstrate that POR promotes ferroptosis by upregulating peroxidation of membrane polyunsaturated phospholipids. A recent study found that COX-2 can regulate ferroptosis through prostaglandins (54). However, Wan Seok et al. (11) used indomethacin (a COX-1/COX-2 inhibitor) to handle BJeLR cells treated with erastin or RSL3 and found that ferroptosis caused by erastin or RSL3 was not affected by indomethacin treatment. This result indicates that COXs play no function in ferroptosis. Therefore, whether COXs have a regulatory effect on ferroptosis needs further study.

## The regulation of iron metabolism

### Autophagy

Degradation and synthesis are tightly regulated in eukaryotic cells due to the continual turnover of substances. There are two main degradation systems in mammalian cells, lysosome (also called autophagy) and the proteasome (55). Autophagy is further divided into three categories, namely macroautophagy, microautophagy and chaperone-mediated autophagy. Since macroautophagy is the most prevalent kind of autophagy, it has been the subject of the most research. Studies have revealed that the regulation of ferroptosis involves autophagy in a complicated way. On the one hand, autophagy can protect cells from ferroptosis by eliminating damaged cellular components and decreasing iron content in cells (56). Additionally, excessive autophagy can promote ferroptosis (57, 58). Here, we introduce the relevant autophagy regulatory molecules and their function in ferroptosis.

### Autophagy associated genes

Our understanding of the process and function of autophagy has considerably improved as a result of the discovery of *ATGs (* 59). So far, more than 40 kinds of *ATGs* have been found in yeast cells. *ATGs* participate in multiple autophagy-activating stages, including initiation, nucleation, elongation, maturation, and fusion (60). Numerous investigations have discovered that autophagy is crucial to ferroptosis (61). In 2016, Daolin Tang et al. (62) found that knockout of *ATG5* or *ATG7* could inhibit erastin-induced ferroptosis, and further studies demonstrated that activation of the autophagy pathway promoted ferroptosis by degrading ferritin. Artesunate can induce ferroptosis in activated hepatic stellate cells, and up-regulation of *ATG3*, *ATG5*, *ATG6/beclin1*, *ATG12* was observed (63). By triggering ferroptosis in clear cell ovarian cancer tissues, superparamagnetic iron oxide nanoparticles (SPIONs) suppress the activity of human ovarian cancer stem cells. Mechanism studies have found that ferroptosis is activated by reducing the autophagy activity of ovarian cancer stem cells. PCR analysis showed that SPIONs treated human ovarian cancer stem cells autophagy-related factors (*ATG3*, *ATG5*, *MAP1ALC3a*, *MAP1ALC3b*, and *MAP1ALC3c*) expression decreased (64). Unfortunately, the authors did not further explore the mechanism by which SPIONs promote ferroptosis, and whether they interact directly with *ATG3* or *ATG5*. The above findings suggest that ferroptosis is the result of the joint action of multiple regulatory factors, rather than being regulated by a single factor.

### Nuclear receptor coactivator 4

*NCOA4* is a protein that plays significant roles in cellular processes related to iron homeostasis and autophagy. Alec C Kimmelman et al. (65) discovered that *NCOA4* was substantially enriched in autophagosomes using quantitative proteomics in 2014. Further research found that *NCOA4* is essential for the transfer of ferritin to lysosomes, and cells missing *NCOA4* are incapable of degrading ferritin. Thus identifying *NCOA4* is a selective cargo receptor for ferritinophagy, which is vital for iron homeostasis (65, 66). Murphy et al. demonstrated that the interaction between *NCOA4* and ferritin was exclusive to ferritin heavy chain (FTH1) specifically (66). By disrupting the *NCOA4-FTH1* protein-protein interaction, it does reduce the amount of iron available to organisms in the cell, thus inhibiting ferroptosis (67). Blocking intestinal *NCOA4* protects against iron overloading in a mouse model of hemochromatosis. And *NCOA4*-mediated intestinal ferritin phagosomes are regulated by *HIF2α (* 68). Because *NCOA4* plays a crucial part in ferroptosis, treating illnesses by targeting *NCOA4* has become an area of intense research (69, 70).

### Iron response element binding proteins

*IRPs* regulate iron metabolism at the post-transcriptional level by binding to iron response elements (*IREs*) of target transcripts, such as TFR1and ferritin H and L subunits (71). Back in the 1990s, researchers discovered that *IRPs* regulate the amount of iron in cells (72). Initially *IRP1* can increase the expression of TFR1and increase cells’ ability to absorb iron (73). Additionally, Galy et al. (74) used Cre/Lox technology to simultaneously ablate *IRP1* and *IRP2* in a tissue-specific manner, and found that *IRP* deficiency significantly reduced *DMT1* mRNA expression. *IRPs* can also regulate ferritin expression. Under iron-deficient conditions, the expression of *IRPs* is increased and the translation of ferritin mRNA is inhibited; while in iron-rich cells, the binding of *IRPs* to the *IRE* of ferritin is reduced (75). Due to its important function in cellular iron homeostasis, its relationship with ferroptosis has attracted much attention in recent years. α-Enolase 1 (*ENO1*) is an essential glycolytic enzyme. Zhang et al. (76) discovered that *ENO1* inhibits mitochondrial ferroptosis by inhibiting *IRP1*, and demonstrated through *in vitro* and *in vivo* experiments and clinical sample analysis that *IRP1* exerts an anti-tumor effect in liver cancer cells by inducing ferroptosis. miRNAs are an essential post-transcriptional regulator involved in numerous physiological and pathological processes. By inhibiting *IRP2* expression, *MiR-19a* inhibits ferroptosis in colorectal cancer cells, thereby promoting the progression of colorectal cancer (77). Studies have shown that elevated iron content in the liver is one of the main causes of liver steatosis. It was found that the levels of *IRP2* mRNA and protein were increased in steatosis liver. Further research has found that *mir-29a-3p* can reduce the expression of *IRP2*, thereby lowering Fe^2+^ levels, thus inhibiting ferroptosis and improving cell damage, according to additional research (78). These studies indicate that modulating ferroptosis at the post-transcriptional level by regulating *IRPs* is conducive to the treatment of diseases.

### Heat shock proteins

*HSPs* are a class of important molecular chaperone proteins, including *HSP110* (*HSPH*), *HSP90* (*HSPC*), *HSP70* (*HSPA*), *HSP60*(*HSPD/E*), *HSP40* (*DNAJ*), and small heat shock proteins (*HSPB*), which are involved in a variety of physiological and pathological processes (79, 80).

*HSP27*, a key component of *HSPB*, protects cells from oxidative stress by inhibiting *TFR1*-mediated iron uptake and decreasing intracellular active iron (81). After ferroptosis was discovered, researchers quickly focused on the function of *HSP27* in ferroptosis due to its critical role in controlling oxidative stress. Inhibiting *HSP27* expression *in vitro* and *in vivo* can increase the anticancer activity of erastin mediated ferroptosis (82). Dihydroartemisinin (DHA) has cytotoxicity to various malignant tumors, but DHA attenuates ferroptosis in glioma cells through the *PERK/ATF4/HSPA5* pathway (83). In pancreatic cancer, *HSPA5* negatively regulated ferroptosis in human pancreatic ductal adenocarcinoma cells. In mechanism, *HSPA5* reduces *GPX4* protein degradation and subsequent lipid peroxidation, thus limiting the anticancer activity of gemcitabine (84). In addition, Zhou et al. (85) demonstrated that overexpression of *DNAJB6* in esophageal cancer can promote the degradation of *GSH*, downregulate *GPX4*, enhance lipid peroxidation, and promote ferroptosis. Additionally, researchers have discovered that *HSP90* and *HSPA8* can promote *GPX4* degradation via autophagy, thus promoting ferroptosis (86, 87). This indicates that the *HSP* family regulates ferroptosis through multiple pathways. More avenues may be discovered in the future.

The activation of heat shock factor 1 (*HSF1*) is triggered when cells are driven by external causes such as nutritional shortage, hypoxia, oxidative stress, and other similar conditions (88). Upon activation, *HSF1* will translocate to the nucleus, thereby facilitating the transcriptional upregulation of HSPs (89). Since *HSPs* are directly regulated by *HSF1*, *HSF1* plays a regulatory role in ferroptosis by regulating HSPs (90). Sun et al. (82) were the first to describe the regulatory role of the *HSF1-HSPs* signaling axis in ferroptosis in 2015. The researchers discovered that the suppression of the *HSF1-HSPB1* pathway resulted in an augmentation of erastin-induced ferroptosis (82). A recent study discovered that *HSF1* enhances prostate cancer cell resistance to ferroptosis by modulating *HSPE1*, and that *HSF1* knockdown can promote prostate cancer cell sensitivity to RSL3 therapy (91). Furthermore, inhibiting *HSF1* specifically can reverse the ferroptosis resistance generated by *GPX4* suppression and greatly improve the *in vitro* sensitivity of resistant cancer cells and tumors to ferroptosis (92). Thus, it may be concluded that *HSF1* is a promising target for ferroptosis.

Current researches have only revealed the function of some *HSPs* in iron metabolism and ferroptosis, and further researches on the effects of other *HSPs* are required in the future.

### Poly (RC)-binding proteins

The *PCBPs* family includes five protein members, but current research mainly focuses on *PCBP1* and *PCBP2*. As cytoplasmic iron chaperone protein, *PCBP1* and *PCBP2* bind to ferritin in the body, promoting iron loading into ferritin, thereby increasing the amount of iron loaded into ferritin (29, 93). The study discovered that mice lacking *PCBP1* in hepatocytes exhibited defects in hepatic iron homeostasis, and ferroptosis occurred even in the absence of iron overload (94). In addition, the latest research found that *PCBP1* can also inhibit the activation of ferritin autophagy by binding to *BECN1* mRNA and inhibit the PUFAs peroxidation by restraining the expression of *ALOX15* mRNA (95). As an RNA-binding protein, *PCBP2* is able to bind and stabilize the expression of *SLC7A11* mRNA, inhibiting malignancy ferroptosis and promoting tumor progression (96). In the future, enhancing the expression of *PCBP1* and *PCBP2* in malignant diseases may become a novel strategy for treating cancers.

### Hepcidin

Hepcidin is an iron-regulating hormone secreted by the liver. FPN, which is the only known cellular iron exporter, is found in duodenal epithelial cells, macrophages, and liver cells and can transport intracellular iron into plasma. The discovery of the hepcidin-FPN axis is a significant development in the field of systemic iron homeostasis. Hepcidin regulates FPN expression in two ways: inducing ubiquitination and endocytosis of FPN (97) and blocking FPN from opening the central cavity (98). A recent study revealed that hepcidin binding to FPN is coupled to iron binding, and the affinity of hepcidin increases 80 times in the presence of iron (99). Binding of hepcidin to FPN restricts iron export even if the transporter is in an outwardly open state (99). Subsequently, people began to pay attention to the role of hepcidin in diseases. It has been discovered that cigarette tar can accelerate the progression of atherosclerosis (AS) by inducing macrophage ferroptosis. Further research has confirmed that tar can upregulate the expression of macrophage hepcidin in AS plaques and downregulate the expression of FPN and SLC7A11. And this change can be reversed by ferroptosis inhibitors Fer-1 and DFO (100). Chlorogenic acid (CGA) is an intestinal protective agent. Studies have found that CGA reduces hepcidin production and increases the expression of FPN in the duodenum by inhibiting the liver *IL-6/JAK2/STAT3* pathway, thereby restoring iron homeostasis and inhibiting ferroptosis (101). Current research on hepcidin and ferroptosis focuses primarily on benign diseases; in the future, it will be necessary to strengthen hepcidin research in malignant diseases.

### Nuclear factor erythroid 2-related factor 2

*NRF2* is a transcription factor that is crucial for cellular defense against oxidative stress and iron homeostasis. *NRF2* regulates iron metabolism primarily via three pathways: Inhibiting the release of free iron from ferritin (102) and the expression of hepcidin (103); Promoting the expression of iron-related enzymes such as heme oxygenase-1 (*HO-1*) (104); Downregulating the expression of *DMT1* to reduce iron uptake (105). Because it plays an important regulatory role in iron metabolism, *NRF2* can also regulate ferroptosis. The expression of *NRF2* is regulated by the *p62-Keap1-NRF2* pathway. In 2016, Sun et al. (106) confirmed that activating the *p62-Keap1-NRF2* pathway can inhibit ferroptosis in liver cancer cells. *P62* is an autophagic protein, also known as *SQSTM1* protein. The latest study found that autophagy inhibition can protect HepG2 cells from alcohol induced ferroptosis by activating the *p62-Keap1-Nrf2* pathway (107). Theoretically, because *NRF2* plays multiple functions in iron metabolism, it can theoretically regulate ferroptosis via various mechanisms. Finally, we summarize the mechanisms by which different genes modulate iron metabolism and their influence on ferroptosis (**Table 1**).

**Table 1:** Genes regulating iron metabolism and their regulation of ferroptosis.

Gene	Impact on iron metabolism	Impact on Ferroptosis
*ATGs*	Promoting ferritin degradation (62)	Promoting ferroptosis (63, 64)
*NCOA4*	Promoting the transfer of ferritin to lysosomes (65)	Promoting ferroptosis (66, 67)
*IRPs*	Increasing the expression of TFR1 (73) Increasing the expression of *DMT1 (* 74) Inhibiting the translation of ferritin mRNA (75)	Promoting ferroptosis (76, 77)
*HSPs*	Inhibiting TFR1-mediated iron uptake (81) Reducing GPX4 protein degradation (84) Promoting the degradation of GSH and GPX4 (85, 87)	Ferroptosis can be both promoted and inhibited (83, 87, 93)
*PCBPs*	Promoting iron loading into ferritin (29, 93) Inhibiting the activation of ferritin autophagy (95) Binding and stabilizing the expression of *SLC7A11* mRNA (96)	Inhibiting ferroptosis (94–96)
*Hepcidin*	Inducing ubiquitination and endocytosis of FPN (97) Blocking FPNfrom opening the central cavity (98)	Promoting ferroptosis (100, 101)
*NRF2*	Inhibiting the release of free iron from ferritin (102) Inhibiting the expression of hepcidin (103) Promoting the expression of iron-related enzymes (104) Downregulating the expression of *DMT1 (* 105)	Inhibiting ferroptosis (106, 107)

ATGs, autophagy associated genes; NCOA4, nuclear receptor coactivator 4; IRPs, iron response element binding proteins; HSPs, heat shock proteins; PCBPs, poly(rC)-binding proteins; NRF2, Nuclear factor erythroid 2-related factor 2.

## Regulation of lipid metabolism

### Arachidonate lipoxygenases

*LOXs* are widely distributed in eukaryotes and prokaryotes, and it has received a lot of attention in eukaryotes. *LOXs* are enzymes that non-heme iron (or in some cases manganese) dependent, which can trigger the peroxidation of PUFAs to fatty acid hydroperoxides (108). Although most mammalian *LOXs* catalyze arachidonic acid and linoleic acid, some can also convert α-linolenic acid, eicosapentaenoic acid, and docosahexaenoic acid. There are six functional *LOX* genes in the human genome, designated *LOX5*, *LOX12*, *LOX12B*, *LOX15*, *LOX15B* and *LOXE3*. Multiple subtypes and subunits comprise *LOXs*, which are generalised lipoxygenases. Current research demonstrates that *ALOXs* is crucial for regulating ferroptosis. Microsomal glutathione S-transferase 1 (*MGST1*) is a membrane-bound transferase that not only inhibits apoptosis (109), but further studies have found that *MGST1* reduces lipid peroxidation by combining with *ALOX5*, thereby inhibiting ferroptosis (110). *ALOX15* can catalyze arachidonic acid to generate 15-Hydroperoxyeicosatetraenoic acid (15-HpETE), and 15-HpETE can promote the triggering of cardiomyocyte ferroptosis (111). In addition, the researchers discovered that the downregulation of *ALOX15* expression in gastric cancer resulted in a decrease in ROS production, which ultimately led to a significant inhibition of ferroptosis, fostering tumor growth, and decreasing sensitivity to cisplatin and paclitaxel (112). In summary, *ALOXs* can play a significant role in the occurrence and regulation of ferroptosis by promoting the generation of phospholipid peroxidation products, boosting the production of ROS, and partaking in lipid peroxidation. Furthermore, *ALOXs* system-mediated ferroptosis is also regulated by *P53* (113, 114).

### Long-chain acyl-CoA synthetase

*ACSL* can catalyze the combination of long-chain fatty acids and coenzyme A (CoA) to form long-chain acyl-CoA (acyl-CoA). Acyl-CoA participates in fatty acid metabolism, membrane modification, and numerous other physiological processes. There are five members of the animal *ACSL* family: *ACSL1*, *ACSL3*, *ACSL4*, *ACSL5*, and *ACSL*6. According to the current research, *ACSL3* and *ACSL4* are capable of regulating ferroptosis (115). Oleic acid protects melanoma cells from ferroptosis in an *ACSL3*-dependent manner, thereby facilitating melanoma metastasis (116). Similarly, in triple-negative breast cancer cells, the secretion of oleic acid from adipocytes increased in the presence of *ACSL3*, which can inhibit lipid peroxidation and ferroptosis in breast cancer cells (117). Furthermore, in a mouse model of myocardial infarction, the release of *miR-223-3p*, a platelet-enriched miRNA, was increased. Whereas *miR-223-3p* leads to decreased secretion of stearic acid-phosphatidylcholine in cardiomyocytes by targeting *ACSL3*, and stearic acid-phosphatidylcholine can protect cardiomyocytes from ferroptosis (118). Consequently, *ACSL3* can induce cells to develop ferroptosis resistance. In contrast, *ACSL4* is a positive regulator of ferroptosis. In a study, Sebastian et al. found that in *ACSL4* KO cells, lipid peroxidation was not detected even when cells were treated with RSL3, indicating that *ACSL4* can promote ferroptosis (119). Phosphatidylethanolamine (PE), containing arachidonic acid (AA) and adrenic acid (AdA), was found to be the preferred substrate for oxidation by lipidomics approach, catalyzting the formation of Acyl-CoA, and then activates the corresponding fatty acids for lipid peroxidation (119, 120). Furthermore, *ACSL4* levels are elevated in the livers of NAFLD patients, thereby promoting the fatty acid β-oxidation capacity of hepatocytes with a goal to reduce fat buildup (121). And studies have found that inhibition of fatty acid β-oxidation can induce ferroptosis (122). These findings suggest that *ACSL4* can regulate ferroptosis through fatty acid β-oxidation. Due to the different catalysis of *ACSL3* and *ACSL4* within cells, they play opposite roles in ferroptosis.

### The membrane-bound O-acyltransferase

*MBOAT* family is a group of genes that encode proteins playing crucial roles in lipid metabolism (123). These enzymes are involved in various aspects of lipid processing and modification within cellular membranes. According to the latest studies, *MBOAT1* and *MBOAT2* can inhibit ferroptosis (124). Jiang et al. (124) used genome-wide CRISPR activation screening technology to demonstrate that *MBOAT1* and *MBOAT2* can selectively convert MUFAs into lyso-phosphatidylethanolamine (lyso-PE), thereby reducing the content of intracellular PUFAs. Moreover, *MOBAT1* and *MOBAT2* are directly regulated by sex hormones. This discovery not only provides a new target for regulating ferroptosis, but also suggests that sex hormones play an essential role in ferroptosis.

### Lysophosphatidylcholine acyltransferase

*LPCAT* is one of the most important enzymes for maintaining phosphatidylcholine (PC) homeostasis. In fact, *LPCAT* is a subtype of enzyme in the *MBOAT* family (123). It controls phospholipid fatty acyl composition by catalyzing lysophospholipid sn-2 reacylation (125). Currently, four varieties of *LPCAT* have been described in the literature: *LPCAT1*, *LPCAT2*, *LPCAT3*, *LPCAT4*. Lpcat3’s role in lipid metabolism is by far the most evident. *LPCAT3* is widely distributed in the human liver, intestines, and adipocytes (126, 127). *LPCAT3* can regulate intestinal lipid absorption, lipoprotein secretion, and liver fat synthesis to maintain systemic lipid homeostasis. In a 2018 study, researchers found that knockdown of *LPCAT3* in 3T3-L1 preadipocytes can reduce levels of polyunsaturated phospholipids and reduce the expression of genes related to fat production (128). In subsequent years, people began to pay attention to whether *LPCAT3* has a regulatory effect on ferroptosis due to the antioxidant properties of polyunsaturated phospholipids. In 2022, Benjamin F Cravatt et al. (129) found that inhibiting the expression of *LPCAT3* can inhibit the occurrence of ferroptosis. Researchers discovered in a recent study that *LPCAT3* can promote the esterification of PUFAsinto phospholipids, provide raw materials for lipid peroxidation, and then promote ferroptosis (130). Furthermore, it has been established that *ZEB* promotes *LPCAT3* transcription in a *YAP*-dependent manner (130). And *YAP* can also modulate the expression of the *ACSL4 (* 131). The above research results suggest that regulating the expression of *LPCAT3* and *ACSL4* by targeting *YAP* expression, thus regulating ferroptosis, may be of great value in disease treatment.

### P53

Since its discovery in 1979, *P53*, a tumor suppressor gene, has been extensively studied for its role in tumors. *P53* is essential for maintaining genome integrity, modulating cell cycle, and inhibiting angiogenesis (132, 133). In the past period, academics have discovered that *P53* can regulate not only cell necrosis (134), apoptosis (135), and autophagy (136), but also ferroptosis (137). Complex signaling pathways are involved in the regulation of *P53* on ferroptosis, and the results of distinct signaling pathways are inconsistent.

Already in 2015, researchers found that P533KR, a *P53* acetylation deficient mutant, lost the function of regulating cell cycle, aging and apoptosis, but retained its tumor inhibitory function and the ability to regulate the expression of metabolic targets. It can inhibit cystine uptake and make cells sensitive to ferroptosis by inhibiting the transcription of SLC7A11 (a component of cystine/glutamate reverse transporter) (15). In recent years, it has also been discovered that *P53* can block the expression of SLC7A11 in conditions such ovarian cancer (138) and acute lung injury (139). In the metabolism of polyamines, *SAT1* (spermidine/spermine N1-acetyltransferase 1) plays a key role. According to Ou et al. (113), *SAT1* deletion can prevent *P53*-mediated ferroptosis. Mechanistic analysis reveals that *SAT1* induces *ALOX15* expression to enhance ferroptosis (113). Additionally, *P53* can control ferroptosis via *ALOX12* (114). However, the specific regulatory mechanism has not been further explored. In a recent study, researchers found that vitamin K epoxide reductase complex subunit 1 like 1 (*VKORC1L1*) is a direct transcriptional target for P53 (140). The *VKORC1L1* enzyme facilitates the synthesis of vitamin K hydroquinone, which is a completely reduced variant of vitamin K that effectively removes lipid peroxides (140, 141). And *P53* promotes ferroptosis by inhibiting the expression of *VKORC1L1 (* 140). Glutaminase 2 (*GLS2*) is a key enzyme in the catabolism of glutamine. Under the action of *GLS2*, glutamine is hydrolyzed into glutamic acid. In 2010, researchers discovered that *GLS2*, as the target gene of *P53*, can increase the content of GSH in cells and reduce the level of ROS, thereby protecting cells from oxidative stress damage (142, 143). The role of *GLS2* in ferroptosis was not considered at the time because the phenomenon of ferroptosis had not yet been discovered. Interestingly, however, in 2015, researchers found that downregulation of *GLS2* could inhibit ferroptosis induced by total amino acid or cystine deprivation (35). Moreover, Suzuki et al. (144) found that both *GLS2*-knockout mouse models and *GLS2*-deficient hepatocellular carcinoma cells confer significant resistance to ferroptosis. Further study of the mechanism found that *GLS2* increases the generation of lipid ROSby promoting the conversion of glutamate to α-ketoglutarate, thereby promoting ferroptosis (144). Obviously, the conclusions of previous studies on the role of *GLS2* in ferroptosis are contrary to the conclusions of recent studies. This phenomenon is worth pondering, and further research is needed to explain this paradox in the future.

The above described *P53* positive regulation of ferroptosis related pathways, and then *P53* expression can also inhibit the sensitivity of ferroptosis. In a 2019 study, Laura D et al. (145) used CRISPR/Cas9 genome editing, small molecule probes, and high-resolution time-lapse imaging techniques to find that *P53* delays the response of cystine deprivation to ferroptosis through transcriptional regulation of *P21*. Therefore, the role of *P53-P21* signal axis in ferroptosis was clarified. Subsequently, Venkatesh et al. (146) demonstrated again in tumor cells that unstable *p53* inhibits the activity and expression of GPX4 by inhibiting *p21* protein. In addition, *P2*1 can also regulate ferroptosis independently of *P53 (* 147). In colorectal cancer, *P53* regulates ferroptosis by regulating the subcellular localization of dipeptidyl-peptidase-4 (*DPP4*) protein. *P53* is able to prevent the nuclear accumulation of *DPP4*. In the absence of *P53*, DPP4 localizes to the plasma membrane and forms a complex with NADPH oxidase 1 on the plasma membrane, thereby increasing lipid peroxidation and iron deposition (148).

Regulation of ferroptosis by *p53* is highly context-specific. Although current studies have shown that *P53* plays an important role in regulating ferroptosis, the specific molecular mechanisms and interrelationships still need further exploration.

### Antioxidant

GPX4, which utilizes reduced GSH as a cofactor to detoxify lipid peroxides as lipid alcohols, thereby inhibiting ferroptosis, is one of the most notable defence mechanisms that cells have evolved to metabolise hazardous lipid peroxides (13). In addition, system x- c, which plays a core role in providing raw materials for the biosynthesis of GSH in cells, is also of concern (149). Therefore, GPX4 and SLC7A11 play a central role in resistance to ferroptosis.

### GSH/GPX4

GSH is a tripeptide found in all mammalian tissues, notably the liver, and is composed of glutamic acid, cysteine, and glycine held together by peptide bonds. Glutamate, cysteine and glycine are synthesized in the cytoplasm of liver cells under the catalysis of glutamate cysteine ligase (GCL) and glutathione synthase (GS) (150). GSH is the most abundant antioxidant in organisms, most of which are distributed in the cytoplasm, and a small part is distributed in the mitochondria and endoplasmic reticulum (151). There are two forms of glutathione in the human body: thiol-reduced (GSH) and disulfide-oxidized (GSSG) (152). Under normal circumstances, the content of GSSH in the human body is very low, and GSH mainly plays an antioxidant role.

GSH is essential for GPX4-catalyzed reactions. There are 8 kinds of GPX4 proteins in mammals, but only GPX4 has the effect of resisting lipid peroxidation (153). GPX4 is a selenocysteine-containing and glutathione-dependent enzyme. By means of GSH, it can catalyze the conversion of particular lipid hydroperoxides into lipid alcohols.

Selenocysteine is the active site of GPX4, and selenocysteine shuttles back and forth between the reduced state (Se-H) and the oxidized state (Se-OH) to complete the biological function. This process is mainly divided into three steps: First, the reduced GPX4(GPX4-Se-H) is oxidized to the oxidized GPX4(GPX4-Se-OH) by lipid peroxides, which are reduced to the corresponding alcohols; Then, GPX4-Se-OH is reduced by the reducing substrate GSH to generate selenium- glutathione adduct (GPX4-Se-SG); Finally, GPX4-Se-SG reacts with GSH again, transforms into GPX4-Se-H, and produces GSSH (13, 153). After the cycle described above, GSH as an electronics body, toxic lipid peroxides are converted into benign alcohols, so GPX4 is known as the gatekeeper of ferroptosis and plays an imperative part in suppressing lipid peroxidation. Finally, electrons are provided by NADPH, and GSSH is reduced to GSH under the catalysis of glutathione reductase (GR). The process of ubiquitination and dephosphorylation of GPX4 has also been found to facilitate the initiation of ferroptosis (154, 155).

### SLC7A11

The synthesis of GSH requires cysteine, and cysteine itself also has reducibility (156). However, extracellular cysteine is highly unstable and rapidly oxidised to cystine (the oxidised dimer form of cysteine) owing to the high level of oxidation in the extracellular environment. Therefore, most cells need cystine transporter system x- c to transport extracellular cystine to the intracellular synthesis of GSH.

**Figure 2:**
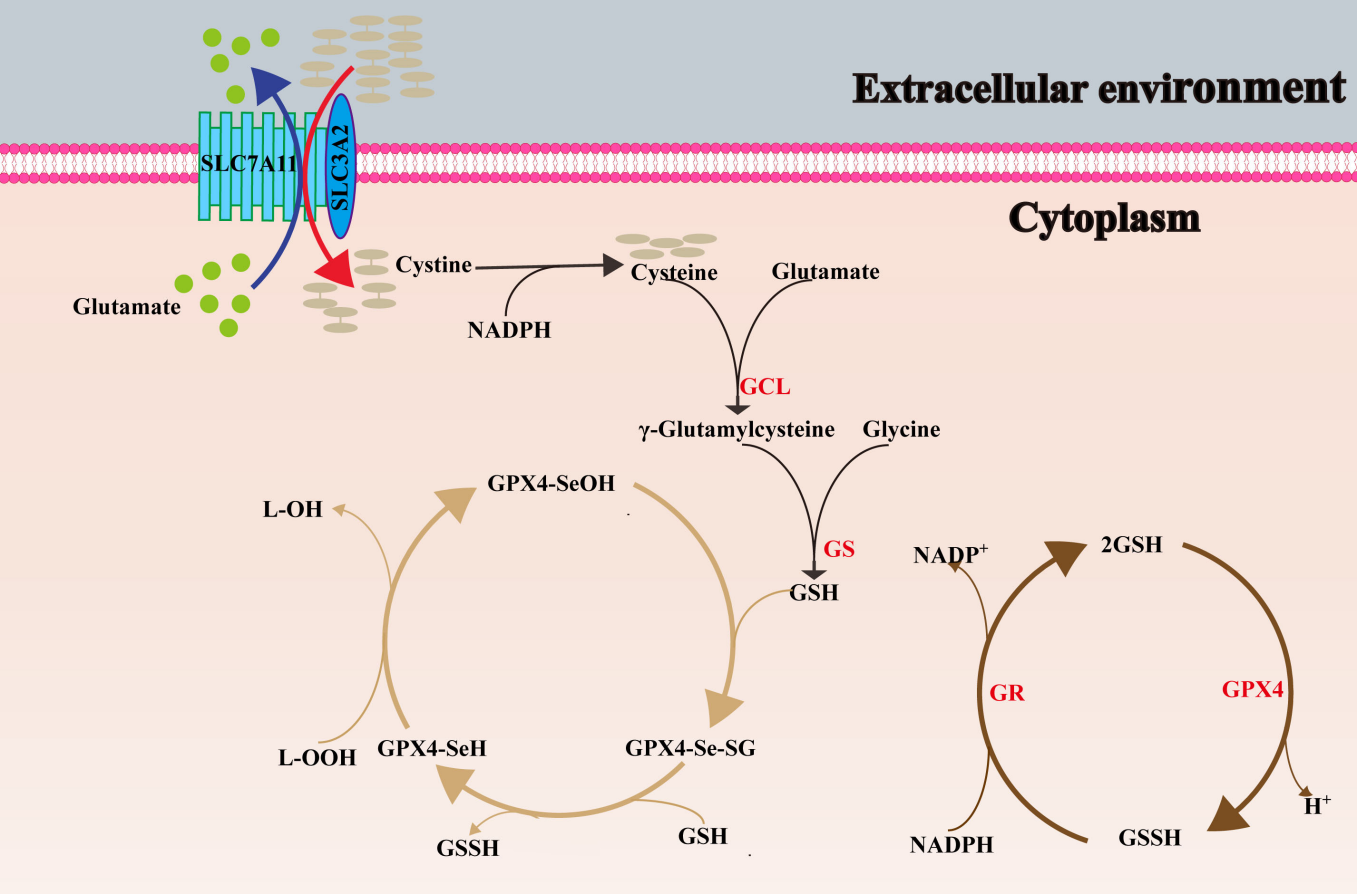
The synthesis process of GSH, and the conversion between oxidized and reduced GPX4. SLC7A11, solute carrier family 7 member 11; SLC3A2, solute carrier family 3 member 2; GCL, glutamate cysteine ligase; GS, glutathione synthase; L–OOH, lipid peroxides; L-OH, alcohol; SeOH, selenenic acid; Se–H, selenol; Se-SG, selenium- glutathione adduct; GR, glutathione reductase.

The system x- c consists of two subunits connected by a disulfide bond, the light chain subunit SLC7A11 (also commonly known as xCT) and the heavy chain subunit solute carrier family 3 member 2 (SLC3A2) respectively. SLC7A11 is a 12-transmembrane protein with both N- and C-termini inside the cell (157). SLC7A11 is highly specific for cystine and glutamate, and is responsible for the main transport work, importing cystine into the cell at a ratio of 1:1 and exporting glutamate outside the cell (149). SLC3A2 is a single transmembrane protein (157), as a chaperone, it is critical to maintain the stability and membrane localization of SLC7A11 protein (158, 159). The cystine transported into the cell is reduced to cysteine by NADPH providing electrons, and then participates in the synthesis of GSH (160). Therefore, the overexpression of SLC7A11 in tumour cells can stimulate GSH synthesis and result in ferroptosis resistance (15). **Figure 2** shows the role of SLC7A11 in GSH synthesis, and the metabolic process of GSH and GPX4 in cells.

### Ferroptosis-suppressor-protein 1

The prevailing perspective in the early stages of research was that GPX4/GSH played a significant role as the primary mechanism for monitoring and regulating ferroptosis. Nevertheless, as research progresses, it has become evident that the responsiveness of various cancer cell lines to GPX4 inhibitors exhibits significant variability (161). This observation implies the existence of other variables that govern the susceptibility of cells to ferroptosis. To verify this result, Sebastian Doll et al. (162) extracted a cDNA expression library from the ferroptosis-resistant cell line MCF7 and screened complementary genes for GPX4 deletion, thereby identifying *FSP1* as an unidentified anti-ferroptosis gene. Subsequent investigation has revealed that myristoylation of *FSP1* is seemingly required for its anti-ferroptotic function (162). Myristoylation facilitates the recruitment of *FSP1* to the plasma membrane (163). Once localized, *FSP1* employs NAD(P)H to facilitate the catalytic regeneration of non-mitochondrial coenzyme Q10 (CoQ10). Ultimately, FSP1 mediates the prevention of ferroptosis by means of CoQ10 (162). In 2019, a groundbreaking discovery was made by researchers, revealing the coordination between the NAD(P)H-FSP1-CoQ10 axis and NAD(P)H-GPX4-GSH in the regulation of ferroptosis (162, 163). Furthermore, in recent years, scholars have made significant progress in the development of diverse small molecule inhibitors targeting *FSP1 (* 162, 164). This achievement has laid a solid foundation for the potential utilization of *FSP1* as a promising therapeutic approach for various diseases.

### Oxidant—ROS

**Figure 3:**
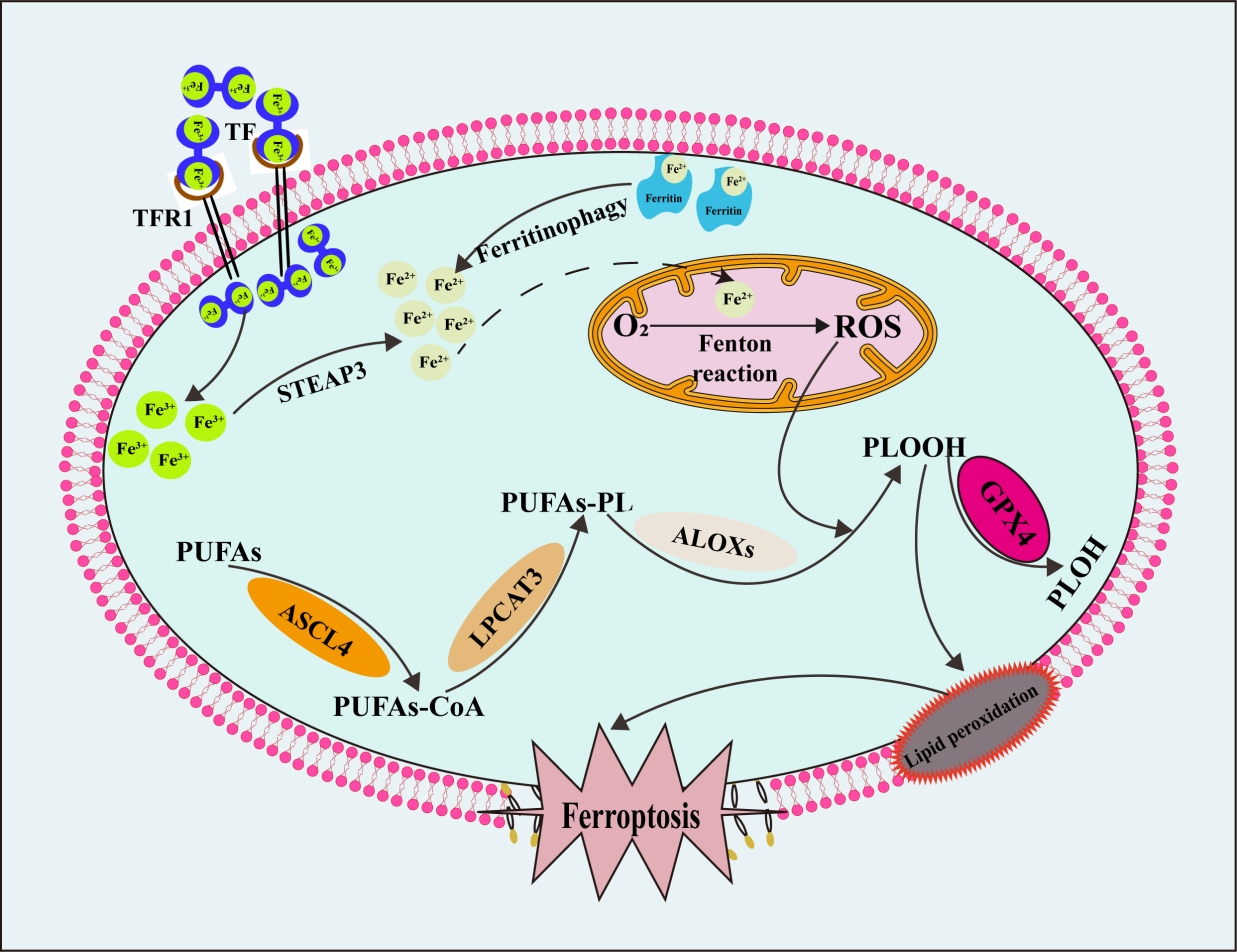
The mechanism and process of ferroptosis. TF, transferrin; TFR1, transferrin receptor 1; STEAP3, six transmembrane epithelial antigen of prostate 3; ROS, reactive oxygen species; PUFAs, polyunsaturated fatty acids; PUFAs-CoA, polyunsaturated fatty acids- coenzyme A; PUFAs-PL, polyunsaturated fatty acids- phospholipid; PLOOH, phospholipid peroxides; PLOH, phospholipid alcohol; ASCL4, long-chain acyl-CoA synthetase 4; LPCAT3, phosphatidylcholine acyltransferase 3; ALOXs, arachidonate lipoxygenases.

ROS are residual products of aerobic metabolism in the organism, including superoxide anion(O_2_^–^), hydroxyl radicals (•OH), peroxyl free radical (ROO•), hydrogen peroxide (H_2_O_2_) and singlet oxygen (^1^O_2_). ROS are produced primarily by mitochondrial metabolism (165) and NADPH oxidase on the cell membrane (166). Appropriate levels of ROS are crucial signaling molecules that play essential roles in signal transduction (167), cellular immune function (168), cell proliferation and cell repair (169). Nonetheless, excessive levels of ROS are toxic, triggering DNA damage (170), inflammatory responses (171), and cell death (172). Lipids dominated by PUFAs are oxidized many times by ROS to produce lipid peroxide, which leads to ferroptosis (13). Therefore, reducing intracellular ROS sources has become a strategy for disease treatment. Finally, as shown in **Figure 3**, we summarized the mechanism and process of ferroptosis.

## Conclusions and perspectives

In the past decade, ferroptosis research has exploded, substantially enhancing scientists’ understanding of the phenomenon. Ferroptosis is the result of the joint action of many biological pathways in the body. The present investigations have unveiled the existence of three primary biological axes that play a crucial role in the regulation of ferroptosis. The first is the biological axis of iron metabolism. The biological axis of iron metabolism encompasses three key components: the regulation of iron absorption, involving proteins such as *IRPs* and *HSPs (* 73, 81); the regulation of iron transport, involving the protein *hepcidin (* 97); and the regulation of iron storage, involving genes associated with autophagy (*ATGs* and *NCOA4*) *(* 62, 65), as well as *PCBPs* and *NRF2 (* 93, 102). The second biological axis involved in lipid metabolism encompasses significant genes such as *ACSL4*, *LPCAT3*, *ALOXs*, *P53*, and the recently identified *MBOAT1* and *MBOAT2 (* 111, 113, 122, 124, 128). Lastly, the antioxidant biological axis encompasses components NAD(P)H-FSP1-CoQ10 axis and NAD(P)H-GPX4-GSH (153, 162, 163). Therefore, we summarized the mechanisms and regulations of ferroptosis from these three aspects. It should be pointed out that even a specific gene can affect multiple links in ferroptosis metabolism. *IRPs*, for instance, can not only promote the expression of *DMT1* and TFR1 (73, 74), but also inhibit the synthesis of ferritin (75), and finally increase the amount of iron in the labile iron pool in cells by acting on three aspects of iron metabolism simultaneously. Among them, the most complex one is *P53*, which can both promote ferroptosis and inhibit ferroptosis through various ways. But the conclusions of the study on *GLS2* are puzzling. In early studies, researchers found that *GLS2* can play an antioxidant role (142). In recent years, studies have found that *GLS2* can promote lipid peroxidation in a variety of cells (144). Different signaling pathways, intracellular microenvironment, and post-transcriptional modification may account for this difference. All of the aforementioned phenomena demonstrate that ferroptosis is regulated by a complex network in cells, and it is insufficient to explain this phenomenon from a single perspective. Furthermore, many studies are limited to the cellular level, but not the *in vivo* level, ignoring the influence of the complex physiological environment and the natural disease development process.

The correlation between ferroptosis and diseases has also been extensively studied. As a potential therapeutic strategy, promoting ferroptosis in malignant diseases and inhibiting ferroptosis in benign diseases has been investigated. Sorafenib is a first-line drug for targeted therapy of hepatocellular carcinoma. Later studies have found that sorafenib can promote ferroptosis in hepatocellular carcinoma (173). The anticancer actions of sorafenib are manifested through the inhibition of the x- c system (174). Additionally, the research investigation revealed a correlation between resistance to sorafenib and cellular resistance to ferroptosis (175). Therefore, in recent studies, researchers have studied the synergistic effect of various chemicals and sorafenib on Ferroptosis, hoping to achieve therapeutic effect by promoting ferroptosis of hepatocellular carcinoma (176, 177). Similarly, in osteoarthritis, researchers found that the occurrence of ferroptosis in chondrocytes promoted the progress of osteoarthritis, so inhibiting ferroptosis in animal models significantly reduced the sensitivity of chondrocytes to oxidative stress, and improved the progress of the disease (178). D-mannose is a monosaccharide naturally occurring in plants. Zhou et al. (179) found that D-mannose reduced the sensitivity of chondrocytes to ferroptosis by inhibiting *HIF-2α*, thereby alleviating osteoarthritis progression and cartilage degeneration. Therefore, the future development of D-mannose-based drugs to treat osteoarthritis will be beneficial for patients. Furthermore, current studies have established that GPX4 and SLC7A11 play an important role in limiting lipid peroxidation; therefore, inhibiting the expression of GPX4 and SLC7A11 in tumor patients can promote tumor ferroptosis in theory. Unfortunately, however, early embryonic lethality was found in GPX4 knockout mice (180, 181), suggesting that in addition to playing a central role in limiting ferroptosis, GPX4 also plays other irreplaceable functions *in vivo*. Although some experiments have achieved ideal results in cell and animal experiments, the translation of theoretical results to clinical practice is still far away.

So far, no specific and rapid monitoring indicators of ferroptosis in living cells or tissues have been found, so one of the urgent problems is to find a gold standard for detecting ferroptosis. BODIPY-C11 and LiperFluo are currently relatively high-accuracy detection methods for detecting intracellular lipid peroxidation (182, 183). Other methods include detection of ferroptosis-related metabolic indicators, such as intracellular Fe^2+^, GPX4, GSH, ROS, MDA, and 4-HNE levels. However, changes in the above indicators may also occur in other types of cell death, such as apoptosis and autophagy (43). Therefore, in actual scientific research work, researchers use a variety of small molecule regulators of cell death to exclude the influence of other forms of cell death. Moreover, different types of cell death may have similar initiators and regulators, for example, GPX4 can also regulate apoptosis and necrosis (184, 185). Moreover, inhibition of ferroptosis may activate other cell death signaling pathways, which ultimately lead to cell death as well (186).

Current research on ferroptosis still faces many challenges and knowledge gaps, but scientists are continually working to improve our understanding of this mode of cell death. With further research and exploration, we can expect a deeper understanding of ferroptosis and the development of relevant therapeutic strategies and applications.

## Author contributions

XZ: Writing – original draft, Writing – review & editing. ZL: Methodology, Writing – review & editing. MW: Methodology, Writing – review & editing. YG: Methodology, Writing – review & editing. XW: Visualization, Writing – review & editing. KL: Methodology, Writing – review & editing. SH: Visualization, Writing – review & editing. RL: Writing – original draft, Writing – review & editing.
